# Diagnosing Cutaneous Melanocytic Tumors in the Molecular Era: Updates and Review of Literature

**DOI:** 10.3390/dermatopathology11010005

**Published:** 2024-01-18

**Authors:** Chelsea Huang, Tiffany Wing-See Lau, Bruce R. Smoller

**Affiliations:** 1 Department of Pathology, Loma Linda University Medical Center, Loma Linda, CA 92354, USA; 2 Department of Pathology, Queen Mary Hospital, Hong Kong, China; lws212@ha.org.hk; 3 Department of Pathology and Laboratory Medicine, University of Rochester Medical Center, Rochester, NY 14642, USA; bruce_smoller@urmc.rochester.edu

**Keywords:** melanoma, molecular, immunohistochemistry, molecular tests

## Abstract

Over the past decade, molecular and genomic discoveries have experienced unprecedented growth, fundamentally reshaping our comprehension of melanocytic tumors. This review comprises three main sections. The first part gives an overview of the current genomic landscape of cutaneous melanocytic tumors. The second part provides an update on the associated molecular tests and immunohistochemical stains that are helpful for diagnostic purposes. The third section briefly outlines the diverse molecular pathways now utilized for the classification of cutaneous melanomas. The primary goal of this review is to provide a succinct overview of the molecular pathways involved in melanocytic tumors and demonstrate their practical integration into the realm of diagnostic aids. As the molecular and genomic knowledge base continues to expand, this review hopes to serve as a valuable resource for healthcare professionals, offering insight into the evolving molecular landscape of cutaneous melanocytic tumors and its implications for patient care.

## 1. Introduction

The exponential growth of molecular and genomic discoveries in the past decade has laid the foundation of the molecular landscape for melanocytic tumors. This unraveled ancillary molecular testing methods and the development of immunohistochemical (IHC) markers toward diagnostic and prognostic evaluations. This article reviews the current genomic landscape of cutaneous melanocytic tumors; briefly provides an overview of the molecular pathways of cutaneous melanomas; and reviews the integration of molecular studies into the differential diagnosis, classification, prognosis, and therapeutic responses of melanocytic tumors. We aim to provide a brief molecular overview and illustrate their practical integration as diagnostic aids.

## 2. Background of the Genomic Landscape

Various genomic studies, including whole-genome sequencing, reveal that the accumulation of multiple genetic alterations contributes to the complex and heterogeneous nature of melanoma development and progression [1]. The genomic landscape of melanoma can vary between individuals and within different subtypes of melanoma. In addition to diagnostic and prognostic purposes, understanding the genomic landscape of melanoma is crucial for developing targeted therapies and personalized treatment approaches. 

### Four-Step Model

**Figure 1:**
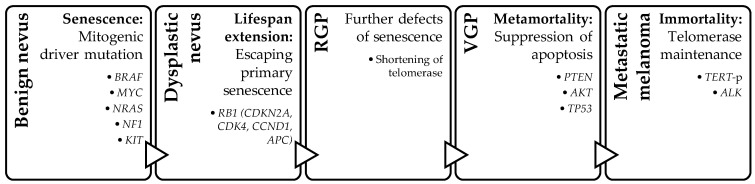
Progression of melanoma and the associated stepwise genetic changes. RGP—radial growth phase; VGP—vertical growth phase.

Several genetic and epigenetic driver changes are identified in melanoma. The complex interplay between genetic alterations and epigenetic modifications contributes to the development and progression of melanoma. The proposed genetic model for melanoma progression includes a four-step model: senescence, lifespan extension, apoptosis suppression, and immortality. Histologically, the corresponding morphologies of this four-step sequence are as follows: benign nevus, dysplastic nevus, thin melanoma/radial growth phase (RGP), invasive melanoma/vertical growth phase (VGP), and metastasis [2]. Not all mutations include all four steps or happen in the above-described sequence; however, these four steps represent the most common sequence in the development of metastatic melanoma [2]. The initial step involves the mitogenic driver mutation in the mitogen-activated protein kinases (MAPKs) pathway, which stimulates cell proliferation by mimicking growth factor signaling. This process includes the interaction of genomic, environmental, and host factors that lead to mutations in *BRAF*, *MYC*, *NRAS*, *ERBB4*, *PTPs*, *NF1*, *KIT*, etc. The second step involves additional mutations that overcome tumor suppressor mechanisms, mainly through disruption of the p16 or RB1 pathway (*CDKN2A*, *CDK4*, *CCND1*, *APC*, etc.), allowing the cells to escape primary senescence and leading to a lifespan extension. The third step involves mutations that suppress apoptosis (*TP53*, *APAF1*, *PTEN*, *PTPs*, *PREX2*, *PTKs*, *AKT*, etc.). The fourth and final step allows the tumor cells to achieve immortality through telomerase reverse transcriptase promoter (*TERT*-p) or *ALK* alterations, which overcome replicative senescence through longer telomeres [2]. (Figure 1).

## 3. Genetic Change: Dysregulation of Three Main Oncogenic Signaling Pathways

In melanomas, the three most frequently dysregulated oncogenic signaling pathways are the MAPK, phosphatidylinositol 3-kinase (PI3K)-AKT, and Wnt/β-catenin signaling pathways. 

### 3.1. MAPK Pathway

The MAPK pathway, also known as the RAS-RAF-MEK-ERK signaling pathway, is a classic intracellular pathway that plays a crucial role in the homeostasis of normal cell turnover, cellular proliferation, differentiation, survival, and apoptosis. Aberrant activation of this signaling pathway induces tumorigenesis and was found to be associated with various malignancies. The activation of the MAPK pathway is via growth factors binding to receptor tyrosine kinases (RTKs) on the cell surface. This leads to the activation of sequential downstream components: Ras, Raf kinases, MEK, and ERK [3]. In melanomas, the hyperactivation of the MAPK pathway is mainly through activating mutations in *BRAF*, *NRAS*, *NF1*, and *KIT* [4]. Alterations in these genes are usually mutually exclusive in untreated melanomas. These alterations can give rise to melanocytic nevi and, therefore, arise early in the progression of melanomas [5]. The most frequently seen primary oncogenic event in melanomas is the *BRAF*-activating mutation, which leads to permanent activation of the MAPK pathway and subsequently promotes uncontrolled cell proliferation, survival, and other oncogenic processes [6,7].

*BRAF* mutations are found in around 50% of all melanomas, with p.V600E being the most common mutation, accounting for 80–90% of *BRAF*-mutated melanomas [8]. Compared with patients without a *BRAF* mutation, *BRAF*-mutated melanomas are seen in the younger age group of <40 years old and in anatomical regions with zero-to-low cumulative sun damage (CSD). Histological characteristics of *BRAF*-mutated melanomas include superficial spreading and/or nodular growth patterns. Other features include pagetoid scatter, nest formation, sharp demarcation, and thickened epidermis. Cytologically, the melanocytes are larger and rounder with more pigment. In contrast, the presence or absence of ulceration, tumor thickness, and mitotic count are not associated with the presence or absence of *BRAF* mutation [9].

Mutations in *NRAS* account for approximately 20% of all melanomas [10,11,12,13]. *NRAS* mutations are associated with an older age group (>55 years) and high CSD, with a preferential occurrence on the upper extremities. Histologically, *NRAS*-mutated melanomas feature thicker primary tumors (greater Breslow depth), greater levels of mitosis, and infrequent ulceration. Prognostically, compared with the *BRAF*-mutated *NRAS* wild-type melanomas, the *NRAS*-mutated melanomas have been related to more aggressive biological behavior with a higher risk of distant metastasis [14]. Of note, *NRAS* is the most common mutation seen in nevoid melanoma, especially the papillomatous type [15].

Also associated with an older age group and high CSD are melanomas with somatic *NF1* mutations. *NF1* mutation is found in 10–17% of cutaneous melanomas [11,13,16,17]. Typically, *NF1* mutations are found in melanomas that lack mutations of *BRAF* and *NRAS*. In contrast to the triple wild-type (*BRAF* wild-type, *NRAS* wild-type, and *NF1* wild-type) melanomas and the *BRAF-* and *NRAS-*mutated melanomas, *NF1*-mutated melanomas have a strong association with ultraviolet (UV) damage, as evidenced histologically by a higher degree of background solar elastosis in the dermis [18]. The *NF1* loss-of-function mutation is found in 45–95% of desmoplastic melanomas (DMs), which is a subtype of melanoma with severe background solar elastosis. DM is associated with a high propensity for local recurrence and occasional regional lymph node spread [19]. Overall, due to the high CSD and the association with a strong UV signature, *NF1*-mutated melanomas are found to harbor a greater mutational burden [20]. The high mutational burden in *NF1*-mutated melanomas demonstrates a favorable outcome for immune checkpoint inhibitor therapy [21].

*NRAS*-mutated melanomas and *NF1*-mutated melanomas are both UV-driven but they are different phenotypically and biologically. An *NF1* mutation is associated with desmoplastic melanomas, while an *NRAS* mutation is associated with nevoid melanomas. *NRAS*-mutated melanomas are aggressive with a high risk of distant metastasis. *NF*-mutated melanomas are associated with local recurrence and regional lymph node spread. 

Activating *KIT* mutations are found in 20–90% of mucosal (anorectal and oral cavity) melanomas, approximately 36% of acral melanomas (AM), and approximately 28% of high-CSD cutaneous melanomas [22,23]. Even though *KIT*-mutated melanomas are found to be closely associated with older age [24], there is still inconsistency regarding the association with other clinicopathological features, such as gender, Breslow thickness, histological types, ulceration, mitotic rate, and tumor stages through different studies [25].

In the MAPK pathway, targeted therapies, such as *BRAF*-inhibitors (e.g., vemurafenib, dabrafenib) and MEK-inhibitors (e.g., trametinib), have been developed to specifically inhibit components of the MAPK pathway in melanomas. These drugs have shown significant efficacy in patients with *BRAF*-mutant melanomas by blocking the excessive signaling and suppressing tumor growth. While targeted inhibition of *BRAF* and MEK has resulted in improved survival in patients with *BRAF* V600E-mutated melanoma, therapeutic resistance is often the end result [26]. The PI3K-AKT pathway plays a significant role in *BRAF*-/MEK-inhibitor resistance in melanoma patients and may represent a crucial target for combination therapy.

### 3.2. PI3K-AKT Pathway

The PI3K-AKT pathway is one of the most important signaling networks in cancer. Activation of this pathway plays a significant role in melanomas, frequently in the setting of concurrent activation of the MAPK signaling pathway through upstream *RAS* oncogene activation [27]. Activation of the *RAS* oncogene in the MAPK pathway leads to the downstream activation of two different but interconnected pathways: the RAF–MEK–ERK pathway and the PI3K-AKT signaling pathway [28]. The PI3K-AKT pathway is activated by various growth factors and cytokines. It involves the activation of PI3K, leading to the production of phosphatidylinositol (3, 4, 5)-trisphosphate (PIP3). PIP3 recruits and activates AKT (protein kinase B), which then regulates multiple downstream effectors involved in cell survival, growth, and metabolism. Dysregulation of the PI3K-AKT pathway in melanomas is commonly observed through mutations or amplifications in genes such as *PTEN*, *PIK3CA*, and *AKT1*. *PTEN* is a negative regulator of the pathway, while *PIK3CA* is responsible for encoding the catalytic subunit of PI3K. Dysregulated PI3K-AKT signaling promotes cell proliferation, survival, and resistance to apoptosis [29].

The common feature in *PTEN*-altered melanomas is the advanced stage at diagnosis. Loss of *PTEN* accumulates throughout the disease as it progresses; hence, the association with advanced-stage melanomas. Some survival analysis studies show that *PTEN*-retained tumors are linked to a better patient outcome, demonstrating the crucial role of this tumor suppressor gene. In contrast, *PTEN* loss is associated with poor survival [30]. Tumor-infiltrating lymphocytes, T-cells, and cytotoxic T-cells are found to be decreased in number and function in tumors with *PTEN* loss. Thus, *PTEN* loss is associated with resistance to immunotherapy in melanomas [31].

### 3.3. Wnt/β-Catenin Pathway

The Wnt/β-catenin pathway is a key component in embryonic development, tissue homeostasis, and stem cell maintenance. Activation of this pathway occurs when the Wnt ligands bind to Frizzled receptors and co-receptors, leading to β-catenin stabilization and translocation into the nucleus. In the nucleus, β-catenin interacts with transcription factors, regulating the expression of target genes involved in cell fate determination and proliferation [32]. Wnt-signaling plays an important function in the skin by guiding the migration of neural crest cells and multipotent precursor cells and driving them toward a melanocyte fate, including terminal differentiation of melanoblasts to melanocytes [33]. In melanomas, Wnt/β-catenin pathway dysregulation leads to aberrant activation of the pathway through mutations in genes such as *CTNNB1* (encoding β-catenin) or *APC*. 

Although dysregulated Wnt/β-catenin signaling promotes cell proliferation, survival, and invasion, the activation of Wnt/β-catenin signaling in melanomas is found to be associated with a lower proliferative index and correlates with a more favorable prognosis [34]. A study on a murine model shows that melanoma cells expressing Wnt3a, a member of the Wnt family, behave similarly to highly differentiated melanocytic cells (high degree of pigmentation, alterations in the cell cycle leading to decreased proliferation, upregulation of melanocytic genes, and formation of smaller tumors) [34]. Another study on mice indicates that Wnt3a expression is linked to decreased metastasis [35]. The murine models reflect the key roles that Wnt3a plays in regulating cell differentiation, proliferation, regeneration, and motility [36].

Wnt-activated deep penetrating/plexiform nevus (DPN) features combined activation of the MAPK and Wnt signaling pathways. Hence, DPN is molecularly defined by the Wnt/β-catenin and MAPK pathway mutations [37]. Mutations of the β-catenin pathway change the phenotype of a *BRAF*-mutated conventional nevus into that of DPN. Demographically, DPN are typically seen in a younger age group as small, sub-centimeter-sized, solitary, dark-pigmented papules/nodules with a predilection for the head and neck region [38]. Histologically, DPN feature symmetrical, well-demarcated, wedge-shaped, dermal-based lesions that extend down into the deep reticular dermis, and sometimes the subcutis. The lesion comprises fascicular or plexiform nests of large uniform epithelioid to spindled melanocytes with low-grade atypia and near-absent mitoses [39]. Despite the deep-seated nature and occasional mild atypia, most DPN have a good prognosis with benign behavior. Both DPN and DPN-like melanomas share activation of the Wnt/β-catenin pathway, suggesting that some DPN can progress to melanoma. Additional molecular oncogenic alterations (*CDKN2A* and *TERT*-p) are necessary for the development of DPN-like melanomas [37]. Morphologically, in addition to the histological features reminiscent of a DPN, DPN-like melanomas show increased cellularity, atypical junctional component with pagetoid spread, poorly circumscribed border and increased depth of lesion, hyperplasia or atrophy of the epidermis, increased tumor cellularity, significant cytological atypia, prominent nuclear pseudo-inclusions, abundant pale cytoplasm, increase mitoses, and associated melanophages [38,40,41]. Other features include ulceration, necrosis, inflammatory reaction, perineural invasion, and lymphovascular invasion [37,42]. Using IHC, DPN and DPN-like melanomas both express β-catenin (cytoplasmic and membranous), cyclinD1 (nuclear and cytoplasmic), and LEF1 [37,43].

These three oncogenic signaling pathways (MAPK, AKT, and Wnt/β-catenin) are interconnected and often coexist in melanomas. They contribute to the dysregulated cellular processes that drive melanoma development, including abnormal cell growth, survival, invasion, and metastasis. 

## 4. Epigenetic Changes: DNA Methylation, Histone Modification, and MicroRNA Dysregulation

While the above-described genetic changes alter the protein end product, epigenetic changes affect gene expression by turning it “on” or “off” and making no changes to the DNA sequence [44]. Epigenetic driver changes further add to the complex interplay between genetic alterations and epigenetic modifications toward melanoma development and progression. The three main epigenetic modifications are DNA methylation changes, histone modifications, and microRNA dysregulation.

### 4.1. DNA Methylation Changes

DNA methylation is an epigenetic modification that involves the addition of a methyl group to DNA molecules, often leading to gene silencing and affecting the expressions of genes involved. It is considered the underlying primary epigenetic mechanism for melanoma development and progression [45]. Numerous genes were found to be involved with DNA methylation in melanomas. Some of those genes include O6-methylguanine-DNA methyltransferase (*MGMT*), *CDKN2A*, Ras-association domain family 1 isoform A (*RASSF1A*), TBC Domain Family Member 1(*TBC1D16*), Platelet-Derived Growth Factor D (*PDGFD*), Thyroid Hormone Receptor Beta (*THRB*), and Zinc Finger E-Box Binding Homeobox 1 (*ZEB1*) [46].

### 4.2. Histone Modifications

Histones are proteins that help to package DNA in the nucleus, and they play a role in gene regulation. Chromosomal DNA is packed into nucleosomes with DNA enveloped around histone protein complexes, which consist of subunits named H2A, H2B, H3, and H4. Modification of the histone subunits either activates or silences gene transcription. Histone subunit methylation and acetylation regulate genetic expression by controlling the DNA accessibility to the transcriptional process and through the participation of additional protein complexes [47]. Abnormal histone modifications, such as acetylation, methylation, or phosphorylation, can impact gene expression and contribute to melanoma development. For example, the loss of histone variant macroH2A, which is generally considered to be transcriptionally repressive, promotes melanoma progression [48]. The overexpression of histone variant H2A.Z.2 promotes cell cycle progression in melanomas and is associated with poor prognoses [49,50].

### 4.3. MicroRNA Dysregulation

MicroRNAs (miRNA) are small non-coding RNA molecules that play a role in regulating gene expression by binding to target messenger RNAs (mRNAs). The dysregulation of miRNAs observed in melanomas is associated with tumor growth, invasion, and metastasis [51,52,53]. Although there is still limited data on miRNA expression profiles in melanomas, molecular techniques, such as quantitative in situ hybridization (qISH) for the fluorescent detection of candidate miRNAs, reverse transcription quantitative real-time polymerase chain reaction (RT-qPCR), and miRNA microarray, have been used to assess different miRNA expression levels in melanomas by comparing normal melanocytes with benign melanocytic lesions. miRNA expression levels can also be compared between primary and metastatic melanomas [54]. In one study that compared primary melanomas to metastatic melanomas, a low expression of miR-203 through DNA methylation was observed in metastatic melanomas, and it was also associated with poor survival. It was suggested that hypermethylation of the miR-203 promoter is a potential mechanism of tumor metastasis [55]. Several studies suggested the role of miRNAs in regulating sensitivity to *BRAF*-targeted therapy [56,57].

## 5. Practical Molecular Knowledge for a Diagnostic Surgical Pathologist

This section reviews the updated available molecular IHC stains and molecular tests that can assist in diagnosis, monitor prognosis, and assess therapeutic response.

### 5.1. IHC for Assessment of Molecular Alteration

Although molecular genetic diagnostic techniques are useful, IHC remains the most cost-effective and most frequently performed tool as the next step to aid in differential diagnosis. Currently, the available IHCs that can assess genomic events include VE1, NRASQ61R, ALK, ROS1, Pan-TRK, BAP1, PRKAR1A, β-catenin, LEF1, and p16 [58,59,60]. This IHC section is divided into two parts. The first part briefly reviews each IHC method and the second part elaborates on the application for selected melanocytic lesions (Table 1).

#### 5.1.1. Genomic Events That Can Be Assessed Using IHC

##### VE1

VE1 IHC assesses an alteration in *BRAF* V600E, which is commonly detected in melanocytic lesions in intermittently sun-exposed skin, such as SSM, conventional nevi, dysplastic nevi, and simple lentigo. However, a positive VE1 stain can also be seen in other melanocytic lesions, such as nevoid melanomas, nodular melanomas, pigmented epithelioid melanocytomas (PEMs), *BAP1*-inactivated melanocytic lesions, and acral melanocytic lesions. Careful interpretation of VE1 needs to be based on the foundation of histological features. Additional stains can help with difficult cases. For example, *BAP1*-inactivated melanocytic lesions are positive for VE1 in addition to BAP1 loss, while PEM is positive for both VE1 and PRKAR1A.

For lesions with *BRAF* fusion, such as Spitz lesions, IHC showing VE1-negative staining cannot specifically detect *BRAF* fusions because wild-type *BRAF* can be overexpressed in other melanocytic tumors [61]. Also, because a significant proportion of *BRAF* fusions are the result of inversions or deletions, a fluorescence in situ hybridization (FISH) break-apart probe may not be sufficient to determine the fusion. Next-generation sequencing (NGS) is the test of choice to produce a more reliable result in such cases.

##### NRASQ16R

The most common *NRAS* alteration, accounting for 82.4% of all *NRAS* mutations, was found to be glutamine-to-arginine substitution at position 61 (*NRAS* Q61R) through the largest single institution cohort [62]. The development of NRAS Q16R IHC provides an accurate, rapid, and cost-effective method for detecting the presence of an *NRAS* Q61R mutation in melanomas [63]. Detection of positive IHC suggests melanomas in the high-CSD pathway.

##### ALK, ROS1, and Pan-TRK

Spitz lesions harbor several alterations, including a few that can be assessed using IHC. Pan-TRK IHC identifies *NTRK1*, *NTRK2*, and *NTRK3* alterations. ALK (clone 5A4) [64] and ROS1 IHC identify mutations in *ALK* and *ROS1*, respectively.

##### β-Catenin and LEF1

Uniform strong nuclear staining with β-catenin and/or LEF1 IHCs suggests the diagnosis of DPN or DPN-like melanomas, confirming the altered Wnt/β-catenin pathway that is distinct in this group of lesions. Lesions without alteration in the Wnt/β-catenin pathway show a gradient expression that features intense staining superficially and gradual loss of staining with increasing depth [65]. β-catenin and LEF1 IHCs are useful in differentiating challenging melanocytic lesions that mimic DPN, such as blue nevi and cellular blue nevi.

##### BAP1

*BAP1*-inactivated nevi or melanocytomas harbor bi-allelic inactivation of *BAP1*, which can typically be demonstrated through a loss of BAP1 nuclear expression on IHC [66]. However, this IHC may not be fully sensitive for the detection of all *BAP1* alterations. *BAP1*-alteration can occur through pathogenic missense mutations, resulting in an impaired but not fully inactivated function of BAP1 protein [66].

##### PRKAR1A

A subset of PEMs is characterized by the combination of two molecular alterations, including an initial alteration in the MAPK pathway and a second alteration in the *PRKAR1A* gene [67]. The *PRKAR1A* alteration is via a loss-of-function mutation. Using IHC, a positive expression of VE1, in addition to the loss of cytoplasmic staining of PRKAR1A, is helpful to support the diagnosis of PEM. However, there is a subset of pure PEMs with *PRKCA* fusion instead of loss of function mutation. In PEMs with *PRKCA* fusion, PRKAR1A expression is retained on IHC [61].

##### P16

P16 is a tumor suppressor and splice product of *CDKN2A*. Complete loss of staining in p16 indicates biallelic or homozygotic inactivation of the *CDKN2A* gene that corresponds to the late molecular event in the oncogenesis in advanced cutaneous melanomas [68]. A strong diffuse or mosaic pattern of p16 indicates no alteration in *CDKN2A*. P16 is useful in three diagnostic scenarios: the differential diagnosis of nodal nevi from metastatic melanomas, evaluation of suspicious atypical melanocytic lesions for malignancy, and identification of aggressive phenotype acquired in conventional melanomas. In sentinel lymph node biopsies, p16 block negativity is seen in metastatic melanomas, while p16 mosaic staining indicates nodal nevi. In dermal or nodular atypical melanocytic lesions, the identification of p16 loss reflects the biallelic inactivation that is strongly associated with malignancy. P16 loss can be seen in areas in melanomas with additional inactivation of *CDKN2A*, indicating a more aggressive biology [69,70].

However, several different cohorts from various centers still show variability in the definition of “positive p16” in terms of p16 staining location in the cell (nuclear or cytoplasmic), percentage, and pattern [71]. A reproducible interpretation and criteria that define a positive p16 reactivity for melanocytic lesions still need to be refined.

#### 5.1.2. Selected Melanocytic Lesions

##### Spitz Family

Spitz melanomas and spitzoid melanomas often share similar and overlapping histologic features; however, the underlying molecular alterations are distinct. A “true” Spitz melanoma shares the same molecular alteration as lesions of the Spitz family, namely, kinase fusion or *HRAS* aberration. Driver mutations of a conventional melanoma, such as *BRAF*, *NRAS*, or *NF1*, should not be seen in the Spitz family. In melanomas showing Spitz-like features, the presence of VE1 and/or NRAS Q61R IHC helps to confirm the conventional melanoma pathway alteration in *BRAF* V600E and/or *NRAS* Q61R, respectively. The appropriate classification of such a lesion should be spitzoid melanoma. On the other hand, IHC with positive pan-TRK, ALK, or ROS1 with the absence of VE1 or NRASQ61R signifies a true Spitz melanoma.

##### Deep Penetrating Nevus 

By the WHO definition, DPN is caused by the combination of MAPK and Wnt/β-catenin signaling pathway activations. Alteration in these two pathways can be detected using positive IHC for VE1, in combination with positive β-catenin, respectively.

As mentioned in the previous section, DPN and DPN-like melanomas both express β-catenin, cyclinD1, and LEF1 due to the shared Wnt/β-catenin pathway alteration. The main difference is that DPN-like melanomas often harbor additional genetic alterations, including *TERT*-p mutations and inactivation of *CDKN2A*. While *TERT*-p is analyzed using sequencing, the assessment of *CDKN2A* can be tested using p16 IHC.

### 5.2. Molecular Tests

Molecular assessments for melanomas can be divided into four categories: tests that assess copy number abnormalities, gene expression profiling, mutation analysis, and imaging mass spectrometry (Table 2).

#### 5.2.1. Genomic Copy Number Assessment

Melanomas and benign melanocytic nevi show many overlapping histopathological features and are often misdiagnosed. Using comparative genomic hybridization (CGH), early research discovered significant differences between melanomas and nevi through copy number variations. Melanoma is characterized by an unstable genome with numerous DNA copy number abnormalities (CNA), whereas nevus shows a lack of or very limited CNA [72]. The discovery of the distinct non-overlapping pattern in genomic abnormalities led to the development of diagnostic platforms to differentiate nevi and melanomas using CNA. The two currently available tests are CGH/single nucleotide polymorphism (SNP) array and FISH [60].

##### CGH/SNP/MIP

CGH is a type of DNA microarray-based technique that provides information on genomic alterations (amplifications or deletions) by comparing the DNA copy number changes between a reference DNA sample and a test sample. The visualization of the intensity ratio of the fluorochromes is used to determine the relative gain or loss of tumor DNA compared with the normal reference at each locus [73]. In melanomas, CGH identifies the genetic alterations by comparing the DNA of melanoma cells with normal DNA samples. The recently emerged SNP microarrays can provide allele frequency data in addition to copy number changes. It is useful to identify selected mutations centering specific SNP and detect copy-neutral loss of heterozygosity events [74]. The uses of CGH/SNP arrays provide an opportunity for diagnostic strategies that help to differentiate melanomas from melanocytic nevi using the non-overlapping pattern of chromosomal aberrations and evaluating DNA copy number alterations [75,76]. One of the challenges in performing CGH/SNP arrays in melanocytic tumors is the low DNA quantities in biopsy samples. Most of the formalin-fixed paraffin-embedded (FFPE) tissue from small biopsies yields a low amount of degraded DNA, resulting in insufficiency for most CGH/SNP array platforms [77]. In the recent decade, novel protocols, such as techniques based on molecular inversion probes (MIP), have improved the ability to analyze degraded DNA [78]. The MIP, with a footprint of only 40 bp that targets SNPs, allows for the evaluation of fragmented DNA from FFPE tissue. The advantage of incorporating MIP is the improved signal-to-noise ratio compared with the conventional CGH/SNP arrays. All the unused MIPs and target DNAs are degraded by exonuclease, leaving behind only the probes. The probes are further amplified and hybridized into a microarray [79].

For melanocytic lesions, a CGH/SNP array is used to aid in the diagnosis of histologically ambiguous melanocytic neoplasms. Most melanomas have an unstable genome with multiple segmental DNA abnormalities, whereas the majority of benign melanocytic nevi have no chromosomal aberrations or have isolated chromosomal gains and losses that are not commonly seen in melanomas. The detection of multiple segmental genomic abnormalities on CGH/SNP suggests a diagnosis of melanoma. 

A CGH/SNP array can be used to differentiate spitzoid melanoma from Spitz nevus. The majority of Spitz nevi are found to carry a normal karyotype using a CGH assay; however, 20% of Spitz nevi are found to have an isolated gain in chromosome 11p (*HRAS* locus) [80,81]. This aberration is almost never found in melanomas or other nevi. Another major distinction between Spitz nevus and spitzoid melanoma lies in the difference in their genomic stability [82]. Assessment of an *HRAS* mutation using CGH/SNP is a reproducible method to help in the differential diagnosis between Spitz nevus and spitzoid melanoma [83].

While CGH/SNP’s novel protocols based on MIP allow for the analysis of small or degraded tissue samples, this technique still requires >25% tumor purity to yield reliable results [84]. In addition to tumor purity on the tissue sample, 10 unstained slides are usually required for CGH/SNP array testing. Because of these requirements, cases of superficial or in situ melanocytic tumors with background heavy inflammatory infiltrate and cases with only a few slides available will be unsuitable for CGH/SNP array analysis [85].

##### Fluorescence in Situ Hybridization (FISH) 

Fluorescence in situ hybridization (FISH), similar to CGH, allows for the detection of genetic abnormalities in cancer cells and is used as an alternative by evaluating a limited number of genomic loci for CNAs. The advantage of FISH is that it requires only 1–2 tissue sections and it allows for the direct visualization of tumor cells under a fluorescence microscope. This facilitates the analysis of small lesions with limited tissue samples and lesions with associated heavy inflammatory infiltrates. The disadvantage of FISH is that it is a targeted technique that requires prior knowledge of the specific genetic alterations being investigated to detect them, such as chromosomal rearrangements or gene amplifications [86]. The four-probe FISH assay targeting 6p25 (*RREB1*), 6q23 (*MYB*), *CEP6* (centromere 6), and 11q13 (*CCND1*) helps to differentiate histologically unequivocal melanomas from benign nevi [86,87,88,89]. FISH positivity can assist in diagnosing the “morphologically intermediate” nevoid melanocytic neoplasms [90]. Subsequently, these sets were modified to 9p21 (*CDKN2A*), 6p25 (*RREB1*), 11q13 (*CCND1*), and 8q24 (*MYC*), improving the discriminatory power in differentiating melanomas from nevi [91]. More specifically, 11q13 (*CCND1*) amplification using FISH can be used as a diagnostic marker for histologically undetermined early acral melanoma in situ [92].

The standard four-probe FISH assay should not be applied to tumors for which melanoma arising in blue nevus is a consideration because genomic CNAs typically observed in melanoma arising in blue nevus (*GNAQ* and *GNA11)* are different than those of the typical melanoma pathways [59].

Although the standard four-probe FISH shows high sensitivity and specificity for conventional melanomas, it has a low sensitivity for detecting spitzoid melanomas [93,94]. The most commonly lost genomic region in spitzoid melanomas was found to be chromosome 9p from the CGH database [95]. Hence, the adaptation of a five-probe FISH assay targeting against 9p21 (*CDKN2A*), 11q13 (*CCND1*), and 8q24 (*MYC*) has improved the discriminatory power for spitzoid melanomas [93,96,97]. Tetraploidy, which is commonly seen in Spitz nevi, is another false-positive caveat in the interpretation of FISH due to the increase in absolute signal counts, which incorrectly reflects the relative imbalances in the actual tumor genome.

Compared with CGH, FISH analysis seems to be more cost-effective; however, it has a relatively high false-negative rate in analyzing melanocytic lesions. FISH investigates only 4–6 genomic loci. Mutations, small insertions or deletions, and genomic rearrangements cannot be detected using FISH. Therefore, both positive and negative FISH results need to be analyzed in the proper clinicopathological context [90].

#### 5.2.2. Gene Expression Profiling 

Gene expression profiling (GEP) assists with the diagnosis and prognosis of melanocytic tumors. The steps of GEP include RNA extraction from the FFPE blocks or tape-stripping from the surface of pigmented lesions, reverse transcription to complementary DNA (cDNA), and then amplification via real-time reverse transcription polymerase chain reaction (RT-PCR) [59,98]. All cells on the tumor tissue, including neoplastic melanocytes, stromal cells, and inflammatory cells, are measured for gene expression. Depending on the different GEP tests used, a proprietary algorithm is used to provide a numerical score range corresponding to the likelihood of the tumor being benign, intermediate, or malignant based on the expression of the genes [99]. The overall sensitivity of the tests is between 90–95% with a variable specificity [100,101]. A few commercially available testing platforms include Myriad myPath test and DecisionDx-Melanoma™.

The Myriad myPath test evaluates the expressions of 23 genes, including the 14 genes involved in melanoma pathogenesis and 9 housekeeping genes. The 14 genes are categorized into three components related to cell differentiation, cell signaling, and immune response. More specifically, one gene related to tumorigenesis (PRAME), eight genes related to immune signaling (*CCL5*, *CD38*, *CXCL10*, *CXCL9*, *IRF1*, *LCP2*, *PTPRC*, and *SSL*), and five genes with multifunctional roles (*S100A9*, *S100A7*, *S100A8*, *S100A12*, and *PI3*). The performed test generates an algorithmic myPath score ranging from −16.7 to 11.1. Higher positive scores indicate a higher suspicion of malignant disease. The test report classifies scores as “benign” (−16.7 to −2.1), “indeterminate” (−2.0 to −0.1), or “malignant” (0.0 to +11.1) [102]. The myPath test is an add-on ancillary test to the standard histopathology assessment. The aim of myPath is to aid clinicians in assessing the likelihood of malignancy based on tumor molecular profile, providing valuable information for personalized patient management decisions and further diagnostic evaluation.

DecisionDx-Melanoma™, also known as 31-GEP testing, constitutes a signature of 31 genes, comprising 28 discriminating genes and three control genes. Its primary purpose is to measure the risk of metastasis in individuals diagnosed with stage 1 or 2 cutaneous melanoma and stratify tumors into two risk groups, low (class 1) and high risk (class 2), for developing metastasis within 5 years of diagnosis. DecisionDx-Melanoma™ aims to provide an independent prediction of metastatic risk, independent of current risk assessment metrics (Breslow’s thickness, ulceration status, mitotic rate, American Joint Committee on Cancer stage, and sentinel lymph node biopsy status). This independent risk prediction could potentially guide more aggressive surveillance and treatment strategies for individuals with high-risk stage 1 or 2 disease than they would have otherwise received based on the current risk assessment metrics [103].

The American Academy of Dermatology’s (AAD’s) 2019 guidelines on the management of primary cutaneous melanomas acknowledge GEP’s investigational potential in ambiguous melanocytic neoplasms; however, the AAD emphasizes caution and limited application of GEP as a diagnostic tool. Similarly, for prognostic GEP tests, the AAD discourages the use of routine molecular testing, including GEP, for prognostication purposes until better use criteria are defined. The application of molecular information in clinical management is not recommended outside of research settings [104]. Overall, the integration of GEP into clinical practice still needs further research.

Comparing GEP with genomic copy number assessment methods (CGH and FISH), GEP is helpful for understanding dynamic changes in gene expression. It enables high-throughput analysis, quantitative measurement of gene expression patterns, and the identification of biomarkers and pathways. GEP facilitates the analysis of entire biological pathways and networks by examining how multiple genes are co-regulated [105]. CGH and FISH focus on localized genomic structure and specific genomic regions or individual genes. CGH and FISH do not provide the same breadth of information on coordinated gene expression across the entire genome as GEP does. CGH and FISH focus on detecting structural variations in DNA (amplifications or deletions) and are not as directly suited for identifying biomarkers based on gene expression patterns. Although GEP has its advantages, CGH and FISH are still valuable for certain applications, such as studying genomic structural variations and the precise localization of specific genes.

#### 5.2.3. Mutation Analysis: Telomerase Reverse Transcriptase Promoter 

Telomerase reverse transcriptase (*TERT*) encodes the catalytic subunit of the telomerase enzyme, which plays a role in maintaining the length of telomeres to prevent cellular senescence. Mutations in the promoter region of the *TERT* gene can result in increased *TERT* expression and telomerase activity, contributing to cellular immortality and tumor growth. The presence of *TERT* promoter (*TERT*-p) mutation is assessed using sequencing. The *TERT*-p mutation is not seen in most nevi, but likely occurs early in the evolution of melanomas. Therefore, the *TERT*-p mutation status can potentially differentiate between nevi and melanomas with fairly high specificity, but it shows a low sensitivity for detecting melanoma in some studies. Currently, there is insufficient evidence to make recommendations for testing [60]. In cutaneous melanomas, the presence of *TERT*-p mutations has been associated with lymph nodes and distant metastases [106,107]. It also appears that a combined *BRAF* and *TERT*-p mutation has a synergistic effect on tumor metastasis [108]. 

#### 5.2.4. Imaging Mass Spectrometry 

Imaging mass spectrometry (IMS) is a technology that maps proteasome molecules directly from FFPE tissue sections to simultaneously reveal the spatial distribution of molecular signatures without the requirement for special labels [109]. While many types of IMS technologies have been reported in the literature, the most common type for melanoma assessment is matrix-assisted laser desorption/ionization (MALDI) [110]. MALDI IMS is used to evaluate the distribution of peptides, proteins, DNA segments, and lipids directly from tissue sections with visualization through spatial resolution. MALDI IMS requires minimal diagnostic material from FFPE tissue. In situ analysis only needs one single 6 μm section. For comparison, an additional H&E-stained section is used for histopathological annotation and a serial section is used for mass spectrometry data acquisition [99]. The proteomic differences help with differentiating between malignant melanomas from benign nevi (overall accuracy of 93%). It also has a high sensitivity (97%) and specificity (90%) in differentiating spitzoid melanomas from Spitz nevi [111,112]. In patients with metastatic melanomas, MALDI IMS is used in negative lymph node tissue to identify a set of proteins to correlate with patient prognosis [113,114]. A study using IMS detected greater proteomic changes in *BRAF*- and *NRAS*-mutated melanomas compared with melanomas without the mutations [115]. This discovery helps with classifying melanomas according to molecular subtypes. However, currently, MALDI IMS is primarily used as a research tool in the field of mass spectrometry and molecular imaging, and it is not widely available as a routine diagnostic tool in clinical settings. The price is high, ranging from approximately 380–500 USD per slide, in addition to data analysis of approximately 110–140 USD per hour from the Harvard Cancer Center [116].

MALDI IMS is a promising diagnostic tool to differentiate malignant melanomas from benign melanocytic nevi, offer potential for the improved characterization of clinical tissues, and suggest biomarker candidates for therapeutic purposes. 

## 6. The Pathways of Cutaneous Melanomas

**Figure 2:**
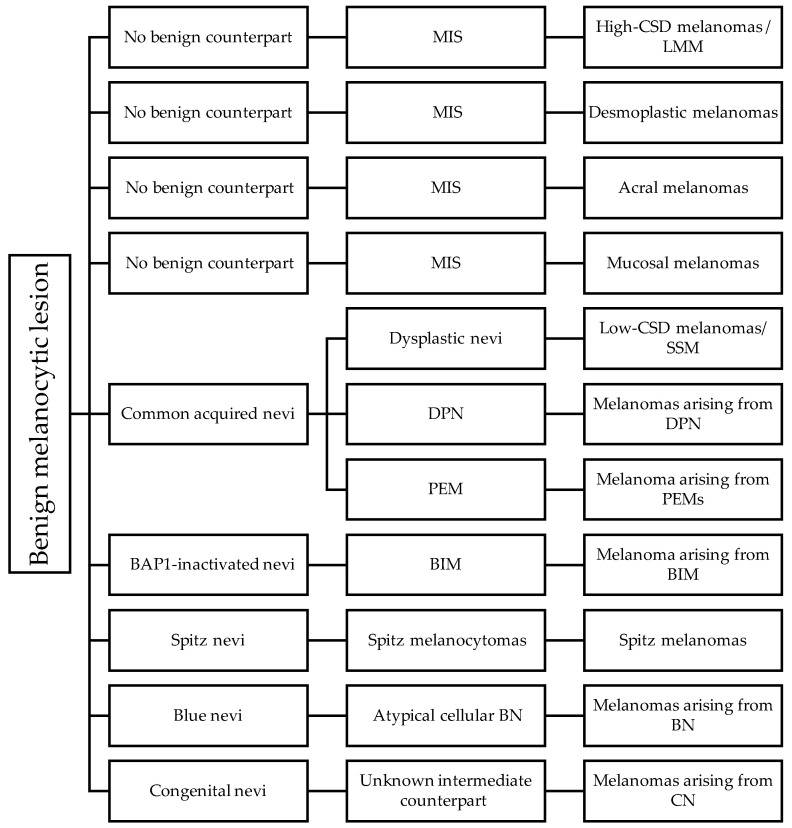
Taxonomy of benign, intermediate, and malignant melanocytic lesions from different pathways. BIM—*BAP1*-inactivated melanocytomas; BN—blue nevus; CN—congenital nevus; CSD—cumulative sun damage; DPN—deep penetrating nevi/melanocytomas; LMM—lentigo maligna melanomas; MIS—melanoma in situ; PEM—pigmented epithelioid melanocytomas; SSM—superficial spreading melanomas.

The current WHO classification categorizes eight pathways of cutaneous and mucosal melanoma development [100,117] (Figure 2 and Table 3).

### 6.1. Pathway 1: Low-CSD/Superficial Spreading Melanoma

The melanoma pathway that is the most encountered and best understood is pathway 1, namely, the low-cumulative-sun-damage (low-CSD) pathway. Low-CSD melanoma is the most prevalent form of cutaneous melanomas, accounting for around 41% of all melanomas [52], most of which present as superficial spreading melanomas (SSM). Both the benign conventional melanocytic nevus and the malignant melanoma of pathway 1 typically harbor a *BRAF* V600E mutation in approximately 80–90% of cases. Melanomas also harbor additional *TERT*-p mutation and CNAs. Low-CSD melanoma is often found within or adjacent to a precursor of conventional melanocytic nevus [59]. When there are borderline features, molecular assessment for the presence of additional mutations, such as *CDKN2A* or *TERT*-p, and chromosomal CNA can be helpful in differentiating low-CSD melanomas from intermediate lesions. The presence of *BRAF* V600E alteration is helpful in differentiating low-CSD melanomas from melanomas of the Spitz lineage.

### 6.2. Pathway 2: High-CSD/Lentigo Maligna Melanoma

Unlike low-CSD melanomas, high-CSD melanomas usually arise de novo and often originate from melanoma in situ. This group usually does not have benign or intermediate/low-grade precursor lesions. High-CSD melanomas occur mostly in elderly individuals, especially in heavily sun-exposed populations, including outdoor workers and persons with frequent recreational sun exposure [118,119]. The most common sites are the heavily sun-exposed sites; 90% of the cases involve the head and neck region [119,120]. The hallmark histological features include a single-cell “lentiginous” pattern of melanocytic proliferation within the epidermis with severe background solar elastosis. A pre-existing nevus is typically absent; melanoma in situ is considered the predominant precursor lesion in this group of melanomas [100,101]. The genomic landscape of high-CSD melanomas differs from non- or low-CSD melanomas and they typically do not harbor the signature *BRAF* V600E mutations. Instead, high-CSD melanoma is characterized by a more miscellaneous set of MAPK pathway mutations, such as *BRAF* V600K, *NRAS*, or *KIT* mutations, or inactivation of the negative regulators of Ras, *NF1*, or *RASA2* [76]. Common specific molecular alterations in this group include bi-allelic inactivating mutations in *NF1* (30%) [121], copy number increases of *CCND1* (20%) [122], activating mutations of *KIT* (10%) [22], inactivating mutations of *TP53* and *ARID2*, and *TERT*-p mutations [119,123,124,125]. The difference in the genomic landscape indicates that high- and low-CSD have distinct genetic profiles that progress through different molecular pathways, and hence, they are different molecular entities. Having predominant UV signature mutations, melanomas of pathway 2 have a very high mutation burden that correlates with their better responsiveness to checkpoint inhibitor immunotherapy [126,127].

### 6.3. Pathway 3: Desmoplastic Melanoma

Desmoplastic melanomas (DM) account for <4% of primary cutaneous melanomas in the population, with a predominant occurrence in pale-skinned individuals [128]. DMs are regarded as a variant of high-CSD melanoma because they commonly arise in skin with a high burden of UV-induced mutations [117,129]. Even though DM harbors overlapping genomic features with high-CSD melanomas, they are independently classified in the 2018 WHO Classification of Skin Tumors due to sufficient and distinctive histopathological features. Histologically, DMs feature a dermal component of spindled, unpigmented melanocytes interspersed between thick, scar-like, collagen bundles; hence, the “desmoplastic” and spindle cell “VGP” patterns. DMs are not necessarily associated with an RGP or in situ component [100,130]. The signature molecular alteration of DMs is the *NF1* bi-allelic inactivating mutation disrupting the “off” state downregulation in the MAPK pathway. Normally, the intact *NF1* function catalyzes the hydrolysis of GTP by *RAS* family members, which accelerates the transition to the “off” state, resulting in the downregulation of the MAPK pathway [131]. Inactivated *NF1* allows *RAS* to remain activated, leading to sustained MAPK pathway activation, thus driving proliferation [132]. Though *NF1* mutations are typically seen in melanomas that lack mutations in *BRAF* or *NRAS*, nearly 4% of melanomas with mutations in *BRAF* or *NRAS* also harbor *NF1* mutations. Conversely, the oncogenic mutations, such as *BRAF* and *NRAS*, frequently found in other melanomas are generally absent in DMs. Because of the shared high-UV signature, *NF1* mutation is also frequently seen in high-CSD melanomas of pathway 2. This showcases the high burden of the UV mutational signature in *NF1*-mutant melanomas [131].

### 6.4. Pathway 4: Spitz Melanoma 

The Spitz family comprises a spectrum of lesions including Spitz nevus (SN), Spitz melanocytoma (atypical Spitz tumor/AST), and Spitz melanoma (SM). Morphologically, these tumors feature distinctive large spindle and/or epithelioid melanocytes with minimal solar elastosis [133]. Spitz tumors are defined as those harboring activating mutations in *HRAS* or kinase fusions by the most recent WHO Classification of Skin Tumors [117].

To date, the kinase component of the fusions found in Spitz neoplasms includes *ALK*, *MET*, *RET*, *ROS*, *NTRK1*, *NTRK3*, *BRAF*, *MAP3K3*, and *MAP3K8* [83,134,135,136,137].

Spitz tumors with *ALK* and *NTRK1* fusions have characteristic morphologic features. Spitz tumors with *ALK* fusions feature a wedge-shaped and plexiform architecture composed of fascicles of amelanotic wavy to fusiform and spindled melanocytes with mild-to-moderate atypia [64,138,139]. *NTRK1*-rearranged Spitz tumors are commonly characterized by wedge-shaped growth of lobulated nests or rosettes of small spindle cells with filigree-like rete ridges and the exaggerated maturation of dermal melanocytes, associated Kamino bodies, and epidermal hyperplasia [140]. This growth pattern is suggested to correlate with a greater dependence on epidermal growth factors compared with non-*NTRK1*-altered Spitz tumors [61].

Spitz melanomas with *BRAF* and *MAP3K8* fusions tend to be epithelioid and high-grade. They often have a sheet-like growth pattern of intermediate to large epithelioid melanocytes with high-grade nuclear atypia and a higher likelihood of showing melanin pigments compared with other fusions [141]. Scattered single cells may be seen within sclerotic stroma [138].

The progression of SN to SM is through the additional genetic inactivation of *CDKN2A* and the acquisition of *TERT-*p mutations [96,142]. *CDKN2A* encodes the p16 protein, which is vital for tumor suppression. The inactivation of *CDKN2A* results in the loss of function in p16, leading to a promoted cell cycle progression. A strong and diffuse mosaic p16 staining pattern indicates SN, as it suggests an intact *CDKN2A* function, and hence, the benign nature of the lesion. In contrast, the complete absence or absence in sizable areas of p16 within the tumor correlates with *CDKN2A* inactivation and is highly suggestive of melanoma [97]. More specifically, the loss of p16 expression indicates a homozygous loss of 9p21, which leads to the alteration of C*DKN2A* [143,144,145]. The intermediate lesion, namely, AST, can present with either the homozygous or heterozygous loss of 9p21, giving rise to variable staining patterns of p16 IHC.

The term “spitzoid” is applied to tumors that morphologically resemble Spitz tumors but lack defining molecular characteristics. In addition, many of these “spitzoid” tumors harbor *BRAF* mutations of the conventional melanoma pathway, and thus, should not fall into the Spitz pathway [130,146].

### 6.5. Pathway 5: Acral Melanoma 

Acral melanomas (AMs) occur on glabrous skin that lacks hair and has a thick stratum corneum. Over the years, AM has been observed to be the most common melanoma subtype in dark-skinned populations (African, Asian, and Hispanic) given the relatively lower incidence of other CSD-related cutaneous melanomas particularly in these darker-skinned populations [147,148,149]. AM comprises over 50% of cutaneous melanomas overall and represents the most common subtype in East Asian countries [150].

It was postulated that traumatic events and mechanical stress contribute to the etiology of AM [151]. Histologically, AMs feature a lentiginous single-cell growth pattern with a broad RGP that may be present for many years before progressing to invasive melanomas. The neoplastic cells often extend along eccrine ducts. Recent studies in mice identified melanocytic precursor cells within eccrine glands, suggesting that the secretory portion of eccrine sweat glands of volar surfaces may be the cell of origin for acral lentiginous melanomas [152,153]. 

AMs have a relatively low burden of point mutations and a high incidence of CNAs with multiple gene amplification, including *CCND1* and *KIT*. The most frequently seen alteration in AMs is *BRAF*, followed by *NRAS* and *TP53* [154]. Although *CCND1* encodes cyclin-D1, the correlation of detecting *CCND1* copy number alteration using cyclin-D1 IHC has not found to be consistent [155]. Therefore, cyclin-D1 IHC cannot be used as a surrogate in place of *CCND1* FISH in melanomas.

### 6.6. Pathway 6: Mucosal Melanoma

Mucosal melanomas (MM), which represents 1–3% of all melanomas, describes melanomas of the head and neck mucosa (oral and nasal cavities), anorectal mucosa, and female genital tract mucosa; rarely, MM can also occur in the lower urinary tract and esophageal mucosa [156,157]. An associated nevus is rarely seen in MM. Histologically, invasive components of MM typically comprise sheets and expansive nodules in the submucosa. [158]. Adjacent intraepithelial or superficially invasive components may be present and usually appear as lentiginous melanocytic dysplasia, melanoma in situ, or RGP. This is a histological clue indicating that a lesion is a mucosal primary [158].

The etiology of MM is still unknown, and it shows no association with UV exposure or carcinogens [159]. Cicaresse et al. showed that HPV and EBV DNA were found in 17% and 20% of mucosal and ocular melanomas, suggesting that these viruses may act as cofactors in the development of mucosal and ocular melanomas [160]. MM has a low mutation burden and is characterized by frequent focal amplifications, deletions, and structural rearrangements, similar to AMs [1,161]. *NRAS*, *BRAF*, *NF1*, *KIT*, *TP53*, *SF3B1*, *CCND1*, *CDKN2A*, and *TERT*-p mutations were described in a substantial portion of MMs of various sites [22,159,162]. *SPRED1* was recently identified as a tumor suppressor in MM and it is often inactivated in the setting of *KIT* mutation [22,162]. Even though MM is known to have a poor prognosis, the diverse driver alterations in MM include some that can suggest potential susceptibility to CDK4/6 and/or MEK inhibitors [159].

### 6.7. Pathway 7: Melanoma Arising from Congenital Nevus

Congenital nevi (CN), especially giant CN, are caused by an alteration of *NRAS* or *BRAF* in the MAPK pathway in utero or shortly after birth [163]. Large CN with satellite lesions have a 10–15% risk of developing melanoma in the patient’s lifetime [164]. Most such melanomas are highly aggressive and they present during the first 5 years of life. Melanomas arising in CN typically appear as a discolored area that looks different than the background CN in terms of color and texture. They present as rapidly growing nodules or plaques with associated ulceration. It is common to see synchronous lymph node metastatic disease at presentation. Histologically, in the prepubescent age group, the principal site of melanoma development is the dermis or subcutis, whereas, in adults, the development of melanoma starts at the dermal–epidermal junction. Frank malignant features, such as expansile growth, necrosis, brisk mitosis, and cytological features, are often seen. 

Giant CN are most frequently associated with *NRAS* mutation while medium-to-small CN harbor *BRAF* V600E as the predominant mutation [165,166]. Up to 95% of giant CN are found to have an *NRAS* mutation and most melanomas arising in giant CN also exhibit *NRAS* mutations [167,168]. Several other alterations, such as *BRAF*, *TP53*, *RAF1*, *PTEN*, *KIT*, *TERT-*p, *CDKN2A*, and *PRKCA*, were also observed in addition to CNAs [169]. Melanomas arising from small or medium CN often harbor an additional *TERT*-p mutation [170]. The genetic alterations characterizing melanoma development in CN remain poorly understood.

### 6.8. Pathway 8: Melanoma Arising from Blue Nevus

Blue nevi (BN) originate from melanocytes that develop from neural crest stem cells [171]. Those melanocytes are not associated with any epithelium. Atypical cellular blue nevus is the intermediate lesion for melanoma arising from blue nevus (MBN). Histologically, the overlying epidermis is usually uninvolved in MBN. The tumor features invasive dense dermal sheets or nodules of large spindled and epithelioid melanocytes that often infiltrate the surrounding adnexal structures. At the periphery of the MBN, pre-existing BN can usually be found. BN do not harbor a UV mutation signature. MBN is caused by somatic mutations activating the Gαq signaling pathway, predominantly in the genes *GNAQ* and *GNA11*, rarely in the upstream receptor *CYSLTR1*, or downstream effector *PLB4* [172,173,174]. *BAP1* and *SF3B1* mutations also contribute to the malignant transformation of BN [175,176].

Loss of nuclear BAP1 IHC strongly supports the diagnosis of MBN in the setting of a suspicious malignant transformation of BN [175].

Distinguishing MBN from other highly pigmented melanomas is often difficult, especially when remnant BN cannot be clearly identified. The presence of an activating Gαq mutation helps to classify lesions into the BN pathway, while the presence of *BRAF* V600E mutation can help to classify lesions into the low-CSD pathway. 

## 7. Discussion

The elucidation of numerous molecular pathways of melanocytic lesions has given rise to a range of ancillary immunohistochemistry and molecular tests based on their respective molecular alterations. These tests serve four main purposes: to differentiate between nevi and melanomas in histologically ambiguous lesions, to classify melanocytic lesions, to predict prognosis, and to predict response to therapy.

For differentiating between nevi and melanomas in histologically borderline lesions, IHC, such as p16, and molecular assays, such as CGH and FISH, may be employed. In addition, *TERT*-p mutation analysis, GEP, and IMS play potential roles in differential diagnosis, but more research is needed before clear recommendations can be made.

To classify melanocytic lesions, IHC, such as ALK, ROS1, and pan-NTRK, are surrogate markers for detecting kinase fusions in Spitz tumors. β-catenin is a surrogate marker for *CTNNB1*-activating mutations in DPN. BAP1 loss is crucial for the diagnosis of *BAP1*-inactivated melanocytic tumors, while PRKR1A1 loss supports the diagnosis of PEM.

For predicting prognosis in melanomas, several GEP assays have been developed for risk stratification.

For predicting response to systemic therapy in high-stage melanomas, *BRAF* mutation status must be assessed, either using IHC specific for the V600E mutant protein or using *BRAF* gene mutation analysis before *BRAF*-inhibitor and MEK-inhibitor therapy can be initiated.

Overall, the role of molecular assays in characterizing melanocytic lesions is expanding, particularly for the purpose of differentiating between benign and malignant tumors. Such diagnostic molecular tests should only be employed on melanocytic lesions in which a definitive diagnosis cannot be reached using histology. Furthermore, their results should be interpreted in the context of the clinical presentation and histologic features and should not overturn the initial histologic impression. The progression from benign to intermediate and finally the malignant counterpart within each molecular pathway is characterized by a gradual accumulation of molecular alterations, and therefore, the molecular assays demonstrate a variable sensitivity and specificity to differentiate between nevi and melanomas for histologically borderline lesions.

## 8. Conclusions

The significant advances in our understanding of multiple molecular pathways in the progression of melanocytic lesions have enabled the development of ancillary molecular tests to aid in their diagnosis, classification, prognostication, and prediction of therapeutic response. Nevertheless, a comprehensive diagnostic algorithm integrating the clinical findings, histological features, and molecular alterations is still in development. In addition, more studies are needed to improve the performance of ancillary molecular tests in the characterization and diagnosis of histologically borderline lesions.

## Figures and Tables

**Table 1:** IHCs with associated genomic events and their role in diagnosing cutaneous melanocytic lesions.

IHC Stain	Molecular Alteration Detected	Types of Melanocytic Tumors in Which It Can Be Found	Role in Diagnosis
BRAF V600E (VE1)	*BRAF* V600E activating point mutation	Acquired nevus Small congenital nevus Melanoma *BAP1-*inactivated melanocytic tumor Deep penetrating nevus	Differentiating between: superficial spreading melanoma with spitzoid morphology (+) vs. low-risk Spitz lesions (−)
NRAS Q61R	*NRAS* Q61R activating point mutation	Large congenital nevus Melanoma	Differentiating between: superficial spreading melanoma with spitzoid morphology (+) vs. low-risk Spitz lesions (−)
*ALK*	*ALK* translocation	Spitz tumor	Diagnosis of *ALK*-rearranged Spitz tumors (+)
*ROS1*	*ROS1* translocation	Spitz tumor	Diagnosis of *ROS1*-rearranged Spitz tumors (+)
Pan-TRK	*NTRK1* or *NTRK3* translocation	Spitz tumor	Diagnosis of *NTRK*-rearranged Spitz tumors (+)
β-catenin/LEF1	*CTNNB1* activating mutation	Deep penetrating nevus Deep penetrating nevus-like melanoma	Diagnosis of deep penetrating nevus (nuclear (+))
BAP1	Loss of function mutation and loss of heterozygosity in *BAP1*	*BAP1*-inactivated melanocytic tumor	Differentiating between: *BAP1* inactivated melanocytic tumor (−) vs. Spitz tumor (+)
PRKR1A1	Loss of function mutation and loss of heterozygosity in *PRKR1A1*	Pigmented epithelioid melanocytoma	Diagnosis of pigmented epithelioid melanocytoma (−)
p16	*CDKN2A* biallelic inactivation	Melanoma	(1) Assist in the diagnosis of dermal and/or nodular atypical melanocytic lesions (atypical Spitz tumor, atypical cellular blue tumor, and atypical proliferative nodule arising in congenital nevus). P16 loss reflects the biallelic inactivation of *CDKN2A* and represents a strong criterion of malignancy. (2) Differential diagnosis between nodal nevus (mosaic staining) vs. metastatic melanoma (block negativity) in the evaluation of sentinel lymph nodes.

(+) positive expression; (−) absence of expression.

**Table 2:** Molecular platforms and application scenarios. CGH/SNP—comparative genomic hybridization/single nucleotide polymorphism; FISH—fluorescent in situ hybridization.

Molecular Platform	Application Scenarios	Pros	Cons
CGH/SNP	Spitz with tetraploidy, superior to FISH	Covers entire genomeHigher sensitivity than FISH	Melanoma infiltrated by other cells can confound the test
FISH	Most helpful for morphologically intermediate lesions CCND1 amplification: early acral melanoma in situ Manage as melanoma if the lesion is positive for *RREB1*, *MYB*, *CEP6*, *CCND1*, or *CDKN2A* FISH, in addition to positive MYC FISH Negative signifies benign	Sparse cellular samples and small samples are sufficient Faster turnaround time Less expensive than CGH/SNP	Need to know the exact foci of translocation or amplification Assess limited area of genome (4–6 genomic positions at a time) False negative: fusion from a small inversion may not be detected False positive: polyploidy, tetraploidy Need experienced personnel for interpretation
Gene expression profiling	Diagnosis and prognosis of melanocytic tumors	Minimal cells required for diagnosis: tape stripping from surface of pigmented lesion is sufficient	Melanoma infiltrated by other cells can confound the test Relatively new, needs more research Expensive
Mutational analysis: *TERT*-p and *BRAF*	*TERT*-p mutation may potentially differentiate between nevi and melanomas *TERT*-p mutation and combined *TERT*-p and *BRAF* mutations are associated with higher risk of metastasis	*TERT*-p mutation has a high specificity for differentiating melanomas from nevi	*TERT*-p mutation has a low sensitivity for diagnosing melanoma
MALDI-IMS	Differentiation between nevi and melanomas Potentially suggest biomarker candidates as therapeutic targets	High sensitivity and specificity Minimal tissue required for assessment	Not readily available in most laboratories

**Table 3:** Classification using the eight molecular pathways and their associated molecular events, useful IHCs, and molecular tests. CSD—cumulative sun damage, DM—desmoplastic melanoma; CN—congenital nevus; MBN—melanoma arising from blue nevus.

Pathways	Low CSD (1)	High CSD (2)	DM (3)	Spitz (4)	Acral (5)	Mucosal (6)	Melanoma Arising from CN (7)	MBN (8)
Initial molecular alteration(s)	*BRAF* V600E *NRAS* *PTEN*	*NRAS* *BRAF*(non-V600) *KIT* *RASA2*	*NF1* *PIK3CA* *NRAS*	*HRAS* Kinase fusion: *ALK*, *NTRK1*, *ROS1*, *BRAF*, *MET*	*CCND1* *KIT*	*CCND1* *KIT* *BRAF*	CNAs *NRAS*: giant CN *BRAF:* medium-to-small CN	*GNAQ GNA11* *CYSLTR1*
Additional molecular alterations	*CDKN2A* *TP53* *PTEN* *TERT*-p	*TP53* *NF1* *ARID2* *CCND1* *CDKN2A* *PTEN* *TERT*-p		*CDKN2A* *BRAF* fusion *MAP3K8* fusion *TERT*-p	*TERT*-p, *CDKN2A*	*NRAS* *NF1* *CDKN2A* *SPRED1* *SF3B1* *TERT-*p	*KIT* *PTEN* *CDKN2A* *TP53* *TERT*-p	*BAP1* *SF3B1*
Useful IHCs	BRAFV 600E (VE1) NRASQ61R p16	NRAS Q61R c-kit	NRAS Q61R	ROS1 ALK pan-TRK p16	p16	p16	NRASQ61R p16	BAP1
Useful molecular tests	Mutation analysis for *BRAF* V600E and *TERT*-p	CGH FISH Mutation analysis for *TERT*-p	Usually not needed	CGH FISH	FISH for *CCND1* amplification	FISH	FISH Mutation analysis for *TERT*-p	FISH
